# Supplementary material for: Transcriptome Shock in Developing Embryos of a Brassica napus and Brassica rapa Hybrid
Source: Int J Mol Sci. 2023 Nov 12;24(22):16238. doi: 10.3390/ijms242216238 (PMC10671433; doi:10.3390/ijms242216238)
Supplement: Supplementary file 1 [file ijms-24-16238-s001.zip › Supplemental_Information.pdf]

Supplemental Information for

Transcriptome shock in developing embryos of the synthesized hybrid between *Brassica napus* and *Brassica rapa*

Weixian Zhou, Libin Zhang, Jianjie He, Wang Chen, Feifan Zhao, Chunhua Fu, and Maoteng Li

Correspondence: limaoteng426@hust.edu.cn

This file includes the following:

Informations of Supplemental Figures S1 to S6

Other supplemental materials for this manuscript include the following:

Supplemental Tables S1 to S7
Supplemental Figures S1 to S6

Informations of Supplemental Figures

Figure S1. Overall evaluation of transcriptomic sequencing data. (A) PCA analysis of gene ex-pression values (FPKM) of all samples. Red dots represent B.rapa, blue dots represent *B.napus*, and green dots represent hybrids. (B) The correlation analysis of the clean reads using the Spearman correlation coefficient. (C) FPKM box diagram of genes in different samples. The horizontal co-ordinate is the sample name, the vertical coordinate is lg (FPKM), and the box plot for each region corresponds to five statistics (top to bottom for maximum, upper quartile, median, lower quartile and minimum).There are three replicates for each sample.

Figure S2. RT-qPCR of differential validations of 12 selected differentially expressed transcripts. Error bars represent the SD for three biological repeats. Experimental data normalized with *ACTIN* genes are shown as blue bars, and relative changes in transcriptomic data are shown as or-ange lines. The depictions of the transcripts and the sequences used for each gene are listed in Table S2.

Figure S3. Expression profile analysis of parents and F1 hybrid. (A-C) KEGG enrichment analysis of parents and F1 hybrid. The size of the circle represents the number of genes enriched to this term, the colour of the circle represents the rich factor. AACC vs. AA, AAC vs. AACC, and AAC vs. AA from left to right, respectively. (D-F) GO enrichment analysis of DEGs of parents and F1 hybrid. Red, green, and blue circles represented the Cellular Component, Biological Process, and Molecular Function. AACC vs. AA, AAC vs. AACC, and AAC vs. AA from left to right, respec-tively.

Figure S4. KEGG analysis of genes associated with embryo development and expression pattern changes of genes in the focal pathways (A-C) KEGG enrichment analysis of genes selected (listed in Table S5). AACC vs. AA, AAC vs. AACC, and AAC vs. AA from left to right, respectively. (D-F) expression profile of DEGs in plant hormone signal transduction pathway. (G-I) expression profile of DEGs in plant-pathogen interaction pathway. AACC vs. AA, AAC vs. AACC, and AAC vs. AA from left to right, respectively. Red squares represent up-regulation of gene expression, green squares represent down-regulation of gene expression, and white squares indicate no significant difference in gene expression.

Figure S5. GO and KEGG enrichment analysis of ELD genes. (A) GO analysis

of genes from A^n-A^r homologous pairs. (B) GO analysis of genes from C^n-A^r homologous pairs. (C) KEGG analysis of genes from An-Ar homologous pairs. (D) KEGG analysis of genes from C^n-A^r homologous pairs. Additivity, A^r-expression level dominance, A^n-expression level dominance, and Transgressive upregulation or Transgressive downregulation from left to right.

Figure S6. Brassinolide synthesis pathway and gene expression pattern. (A) Brassinolide synthesis pathway and expression of key genes in three species. (B) Overall expression patterns of brassinosteroids synthesis genes.
