# Supplementary figures and images for: Transcriptome Shock in Developing Embryos of a Brassica napus and Brassica rapa Hybrid
Source: Int J Mol Sci. 2023 Nov 12;24(22):16238. doi: 10.3390/ijms242216238 (PMC10671433; doi:10.3390/ijms242216238)

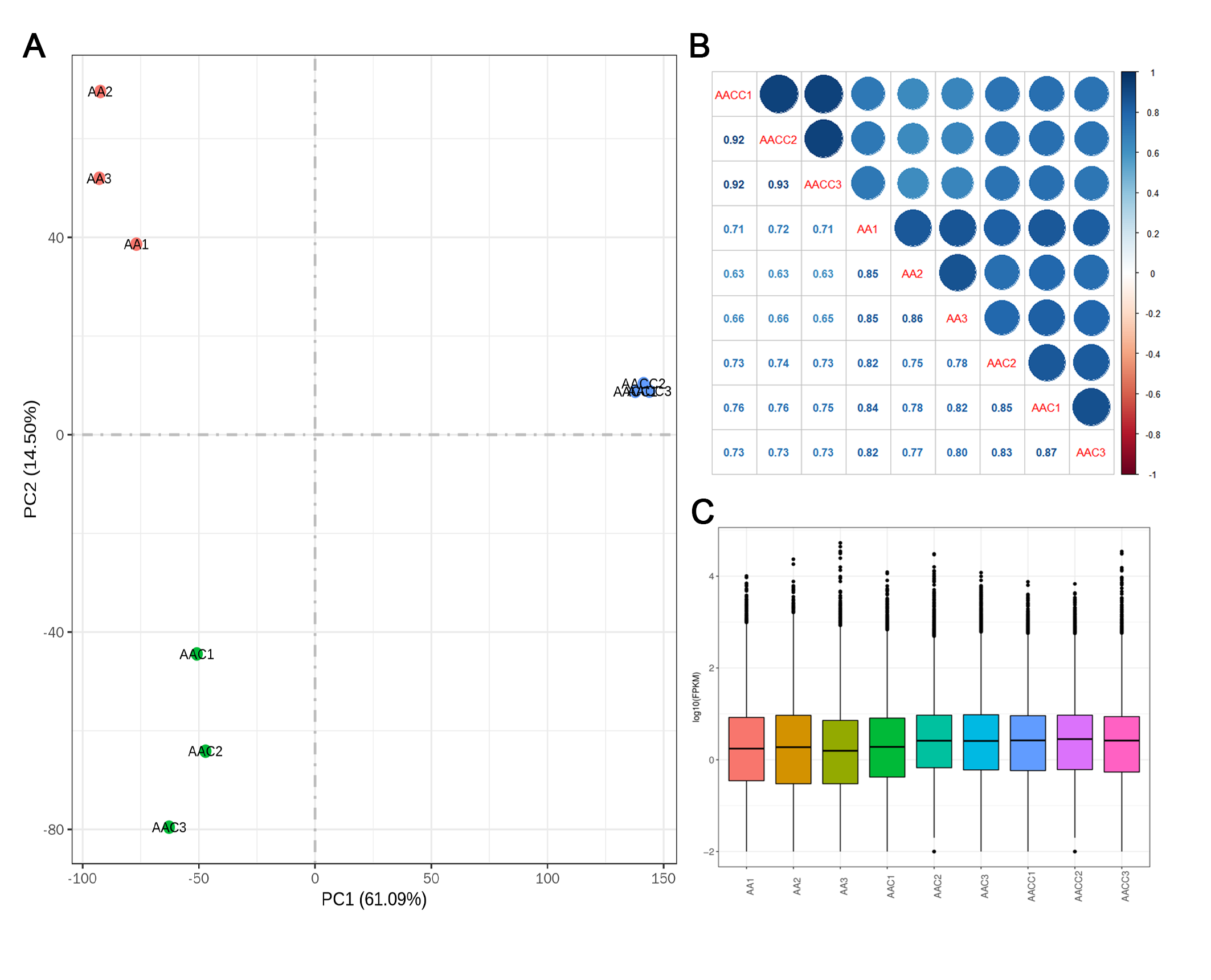

Supplement: Supplementary file 1 [file ijms-24-16238-s001.zip › Supplementary Figures/Figure S1.png]

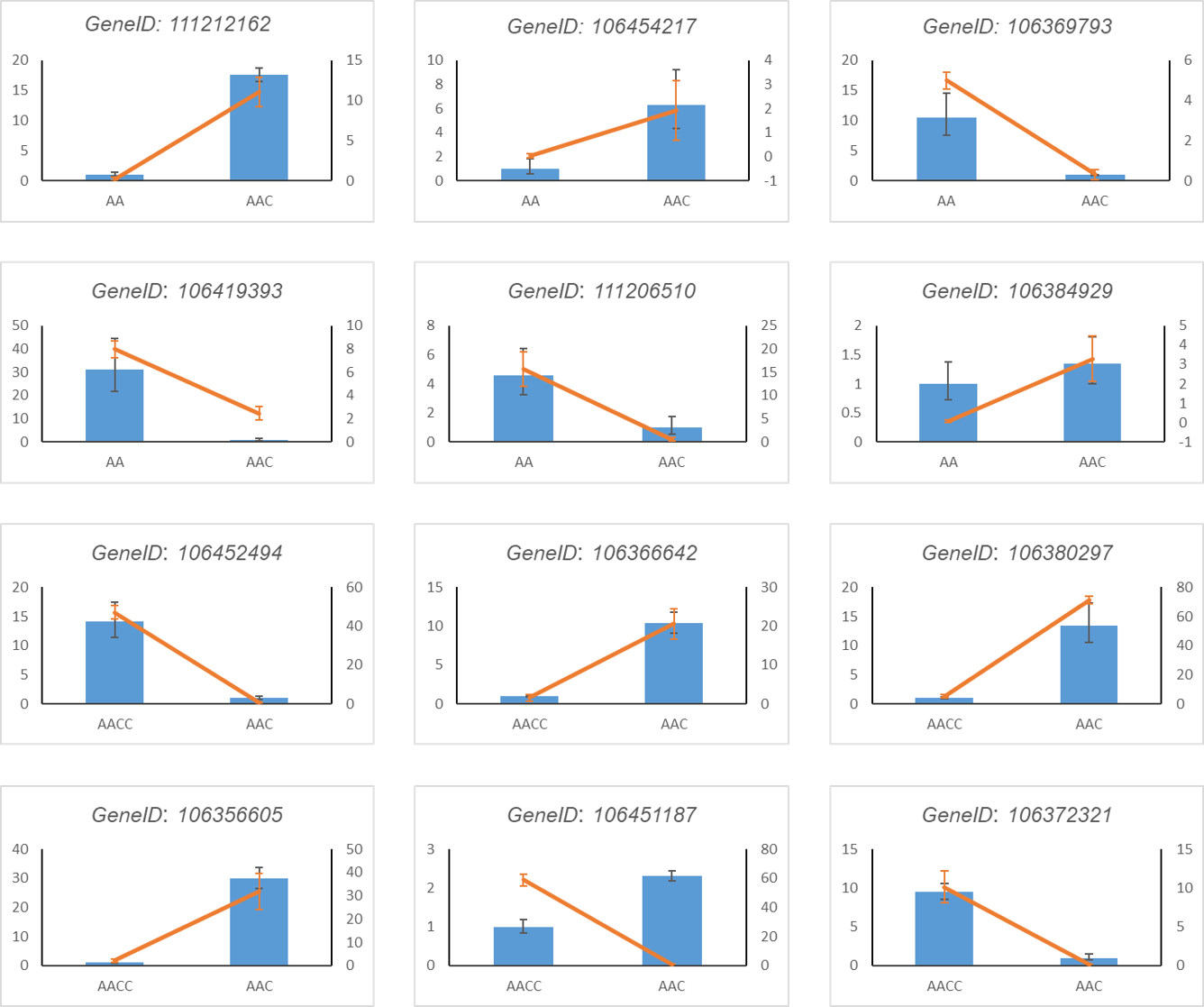

Supplement: Supplementary file 1 [file ijms-24-16238-s001.zip › Supplementary Figures/Figure S2.png]

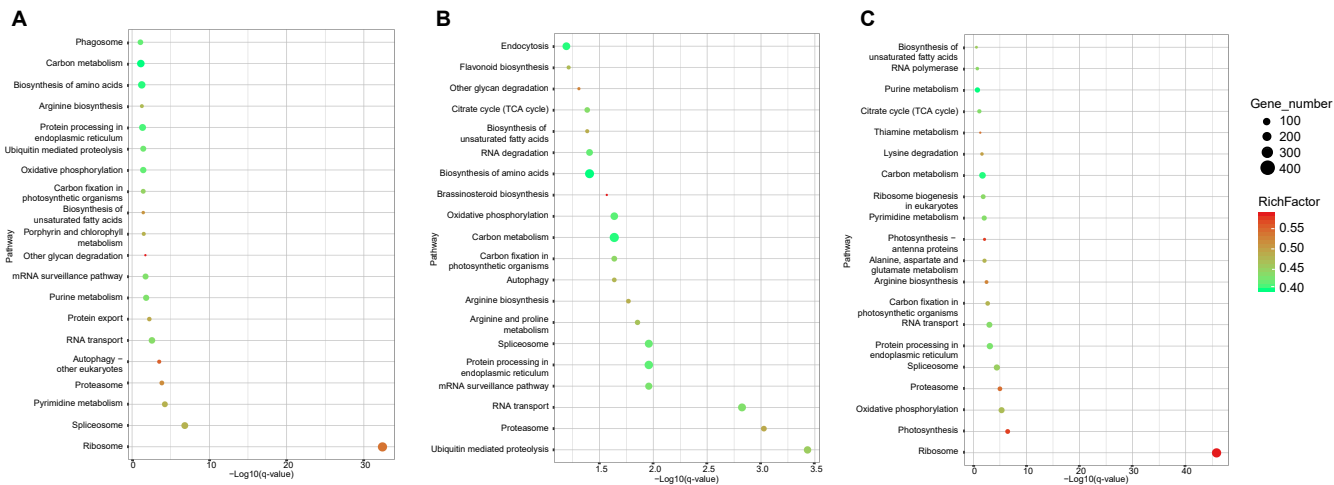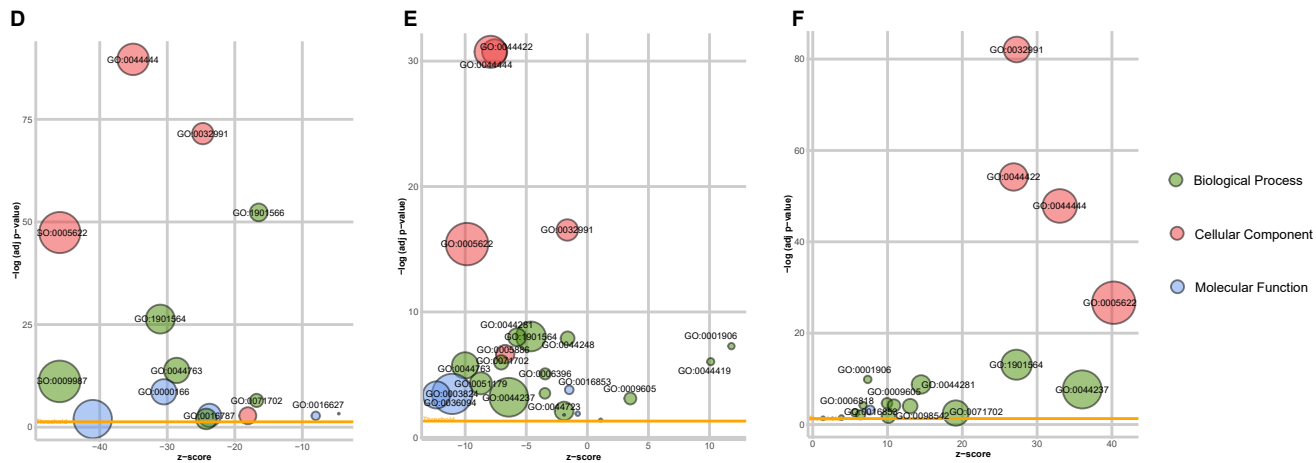

Supplement: Supplementary file 1 [file ijms-24-16238-s001.zip › Supplementary Figures/Figure S3.pdf]

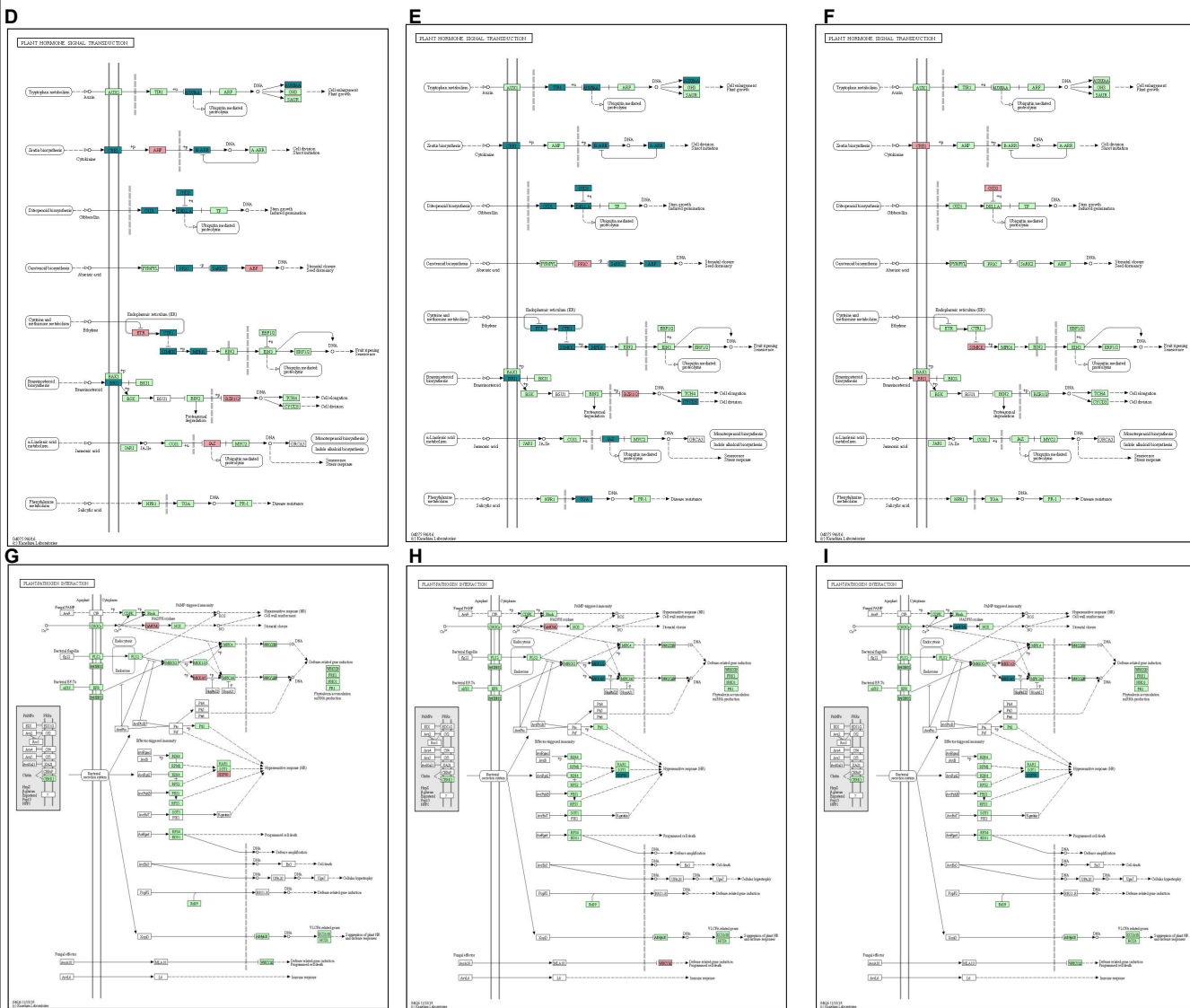

Supplement: Supplementary file 1 [file ijms-24-16238-s001.zip › Supplementary Figures/Figure S4.pdf]

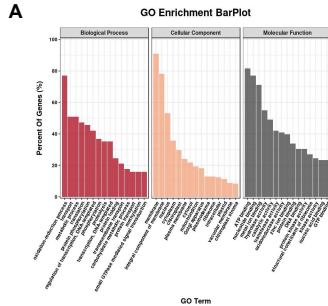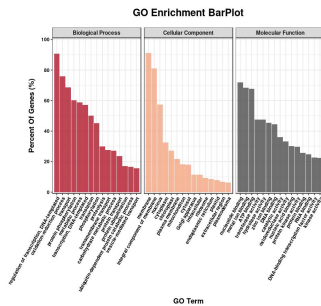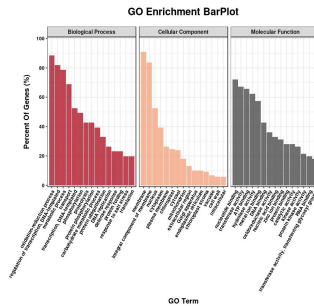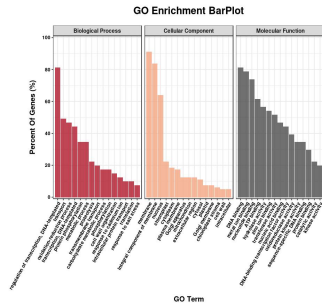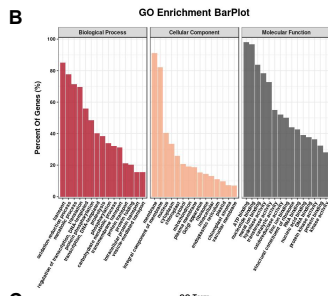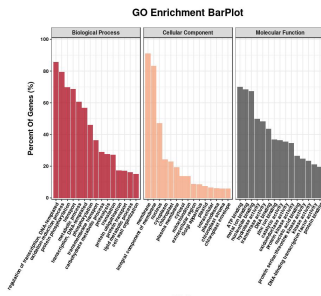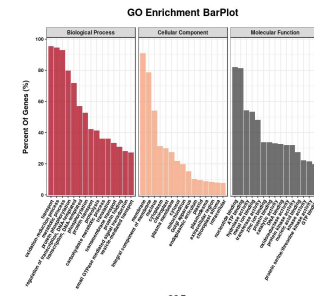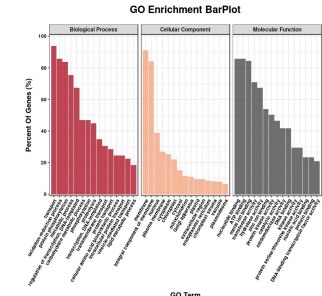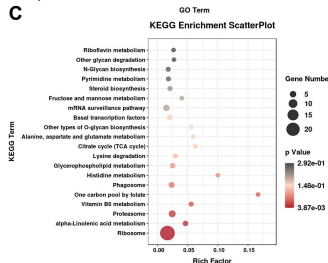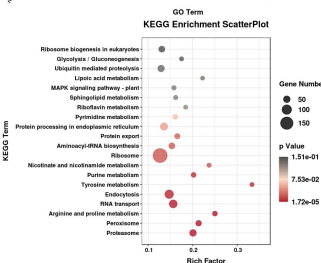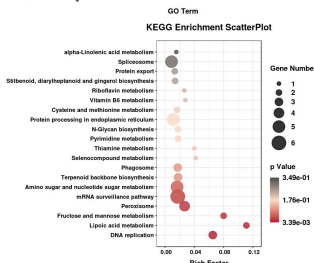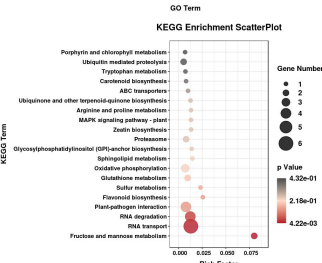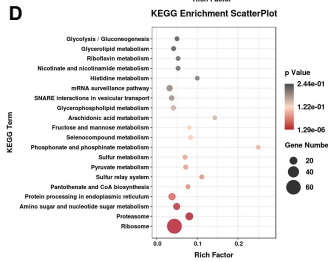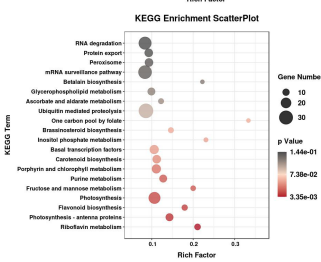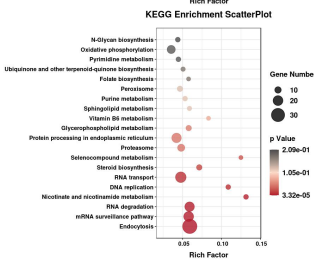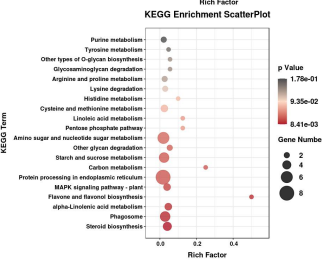

Supplement: Supplementary file 1 [file ijms-24-16238-s001.zip › Supplementary Figures/Figure S5.pdf]

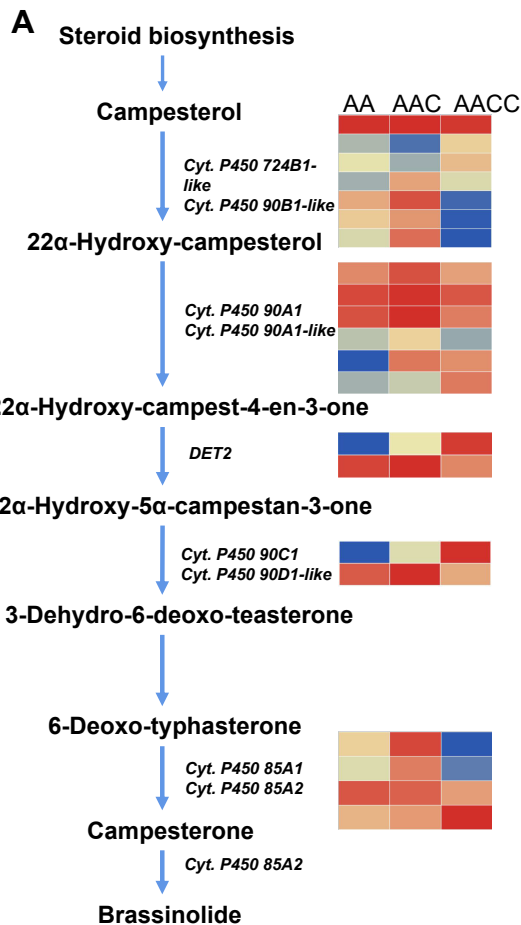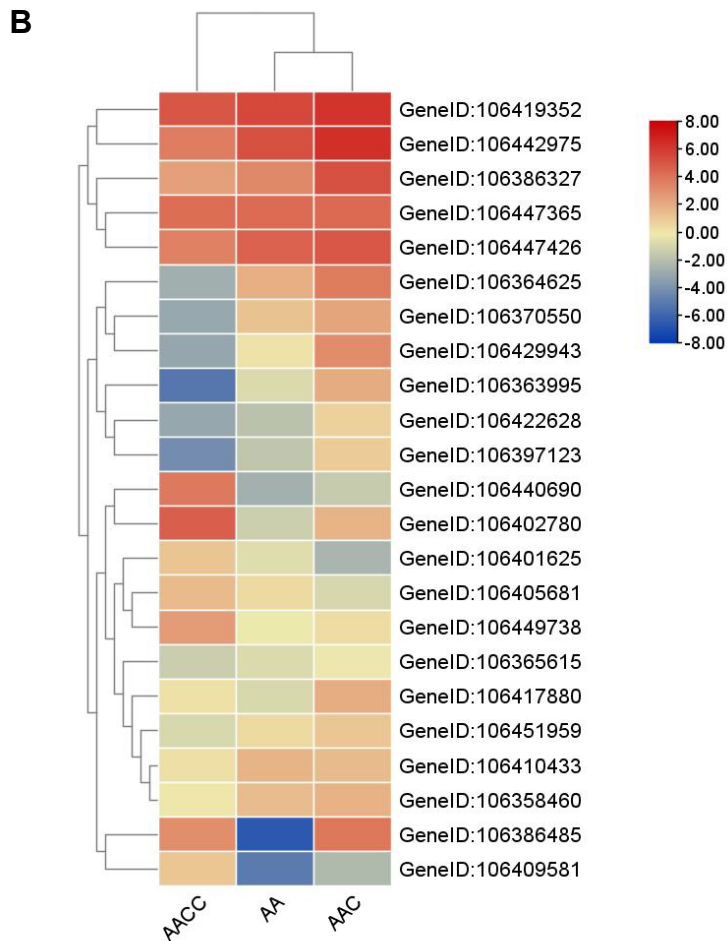

Supplement: Supplementary file 1 [file ijms-24-16238-s001.zip › Supplementary Figures/Figure S6.pdf]
